# Phase II trial of mapatumumab, a fully human agonistic monoclonal antibody that targets and activates the tumour necrosis factor apoptosis-inducing ligand receptor-1 (TRAIL-R1), in patients with refractory colorectal cancer

**DOI:** 10.1038/sj.bjc.6605507

**Published:** 2010-01-12

**Authors:** T Trarbach, M Moehler, V Heinemann, C-H Köhne, M Przyborek, C Schulz, V Sneller, G Gallant, S Kanzler

**Affiliations:** 1 Department of Medicine (Cancer Research), West German Cancer Centre, University Hospital Essen, Essen 45122, Germany; 2 Universitätsmedizin Mainz, Mainz, Germany; 3 Klinikum der Universität München, Klinikum Grosshadern, München, Germany; 4 Klinikum Oldenburg, Oldenburg, Germany; 5 Human Genome Sciences Europe GmbH, Düsseldorf, Germany; 6 Human Genome Sciences Inc., Rockville, MD, USA; 7 Leopoldina Krankenhaus, Schweinfurt, Germany

**Keywords:** mapatumumab, apoptosis, TRAIL-R1, phase 2, colorectal cancer

## Abstract

**Background:**

Recombinant tumour necrosis factor-related apoptosis-inducing ligand (TRAIL) induces tumour-selective apoptosis in various pre-clinical models by binding its specific receptors expressed on cancer cells. Mapatumumab is a fully human monoclonal antibody that is agonistic to the TRAIL Receptor 1 (TRAIL-R1).

**Methods:**

This phase II multicentre study was designed to evaluate the efficacy and safety of mapatumumab in patients with colorectal cancer (CRC) who had failed to respond to, were intolerant to, or not candidates for fluoropyrimidine, oxaliplatin, and irinotecan-based regimens. All patients received two loading doses of mapatumumab (20 mg kg^−1^ every 14 days), followed by maintenance therapy with 10 mg kg^−1^ infused every 14 days.

**Results:**

A total of 38 patients, who had progressive disease after a median of three earlier chemotherapy lines, were enrolled. No response according to the Response Evaluation Criteria in Solid Tumors was observed. A total of 12 patients (32%) achieved stable disease for a median of 2.6 months. The median progression-free survival was 1.2 months. The most common adverse events reported, regardless of relationship, were fatigue, nausea, anorexia, and abdominal pain. Plasma mapatumumab concentrations were within the range of exposures predicted by the results of phase I studies of mapatumumab.

**Conclusion:**

No clinical activity of single-agent mapatumumab was observed in patients with advanced refractory CRC. However, on the basis of its favourable safety profile and pre-clinical evidence of potential synergy in combination with agents commonly used in the treatment of colorectal cancer, further evaluation of mapatumumab in combination with chemotherapy is warranted.

Colorectal cancer is one of the leading causes of cancer morbidity and mortality worldwide, with approximately one million new cases (9.4% of all cancers) and >500 000 deaths observed in 2002 (Parkin *et al*, 2005). About 25% of all colorectal cancer patients have metastatic disease at diagnosis, and although the overall 5-year survival rate for patients with colorectal cancer in North America is 65%, the rate drops to ⩽10% in patients with metastatic disease (Goldberg, 2005).

Standard treatment for patients with metastatic colorectal cancer usually consists of combination therapy of 5-fluorouracil with oxaliplatin or irinotecan. Prospective studies have demonstrated that, in patients with metastatic colorectal cancer, these combinations improve response rates, and progression-free and overall survival compared with treatment with 5-fluorouracil alone (Meyerhardt and Mayer, 2005). The introduction of monoclonal antibodies targeting the epidermal growth factor receptor or the vascular endothelial growth factor has further improved the outcome of metastatic colorectal cancer patients. Thus, cetuximab, panitumumab, and bevacizumab, administered as a single agent or in combination with chemotherapy, have significantly expanded the armamentarium of treatment options (Chung and Saltz, 2005). Nevertheless, the long-term prognosis of these patients remains poor.

The efficacy of cytotoxic anticancer drugs relies on their propensity to trigger cellular effector pathways, such as cell cycle arrest or apoptotic cell death. Apoptosis is a particularly desirable treatment outcome, as it eradicates cancer cells without causing a major inflammatory response, which could provide unwanted survival signals. However, many cancers develop functional defects in the drug-induced apoptosis pathway, which may lead to constitutive or acquired resistance. To this end, alternative pathways, such as the one activated by death receptors including Fas/Apo-1, or tumour necrosis factor-related apoptosis-inducing ligand Receptor 1 (TRAIL-R1) and TRAIL Receptor 2 (TRAIL-R2), are being explored for cancer treatment. The TRAIL-R1 and TRAIL-R2 are expressed in a broad range of solid tumours, including carcinomas of the colon, lung, pancreas, ovary, cervix, uterus, breast, and stomach, as detected by immunohistochemistry. Pre-clinical studies have shown that TRAIL and agonistic antibodies directed to TRAIL receptors can induce apoptosis in cancer cell lines and inhibit tumour growth in xenograft models (Pukac *et al*, 2005; Ashkenazi, 2008; Humphreys and Halpern, 2008; Johnstone *et al*, 2008).

Mapatumumab, a recombinant, fully human agonist IgG_1_ monoclonal antibody that binds with high affinity to TRAIL-R1, shows cytotoxic activity *in vitro*, and inhibits the growth of human tumour cell lines, including colorectal cancer cell lines, in xenograft models *in vivo*. In two phase I single-agent trials, mapatumumab administered at doses ranging from 0.01 to 20 mg kg^−1^ was well tolerated in patients with advanced solid tumours (Tolcher *et al*, 2007; Hotte *et al*, 2008). Adverse events, such as fatigue, fever, and myalgia, were generally mild to moderate in severity. The maximum tolerated dose was not identified at doses up to 20 mg kg^−1^ administered every 28 days. In terms of efficacy, stable disease (SD) was observed in a number of heavily pre-treated patients at several dose levels. Therefore, doses of 10–20 mg kg^−1^ were believed to be safe and potentially effective for further evaluation.

This multicentre phase II study was undertaken to evaluate the efficacy, safety, and tolerability of mapatumumab at a dose of 20 mg kg^−1^ administered every 14 days (cycles 1 and 2), followed by 10 mg kg^−1^ every 14 days in patients with advanced colorectal cancer who had failed to respond to, were intolerant to, or not candidates for a fluoropyrimidine-, oxaliplatin-, and irinotecan-based regimen.

## Patients and methods

### Study design

This was an open-label, multicentre, single-arm phase II trial conducted in four centres. The primary efficacy end point of the study was tumour response defined by the Response Evaluation Criteria in Solid Tumors (RECIST, Version 1.0; Thérasse *et al*, 2000). Secondary efficacy end points were time to response, duration of response, and progression-free survival (PFS). Overall survival was not an end point of the study. Time to response was defined for responders as time from first treatment to first partial response (PR) or complete response (CR), which was subsequently confirmed. Duration of response was defined for responders as time from first PR or CR (which was subsequently confirmed) to disease progression. Progression-free survival was defined as time from first treatment to disease progression or death. The following parameters were also evaluated: frequency and severity of treatment-related adverse events, laboratory parameters, vital signs, plasma mapatumumab concentrations, and mapatumumab immunogenicity. The study was conducted according to the principles of good clinical practice, the ethical principles stated in the Declaration of Helsinki, and the local legal and regulatory requirements. The protocol was approved by the institutional review board at each study site. All patients gave written informed consent.

### Patient selection

Patients with relapsed or refractory histologically or cytologically confirmed locally advanced or metastatic colorectal adenocarcinoma not amenable to intervention with curative intent were eligible for the study. Patients had to have failed, be intolerant to, or not be candidates for fluoropyrimidine, oxaliplatin, and irinotecan-based regimens. Monoclonal antibody treatment including investigational monoclonal antibodies was not allowed within the last 3 (murine or chimeric) or 8 weeks (human or humanised). Eligibility criteria also included age ⩾18 years; an Eastern Cooperative Oncology Group (ECOG) performance status of 0 or 1; measurable disease; no major surgery, radiation, chemotherapy, or investigational agent within 4 weeks; adequate organ function (absolute neutrophil count ⩾1.5 × 10^9^ l^−1^; platelet count ⩾100 × 10^9^ l^−1^; aspartate transaminase (AST) and alanine transaminase ⩽2.5-fold the upper limit of normal (ULN) and ⩽five-fold of the ULN if liver metastases were present; total bilirubin ⩽1.5-fold of ULN; serum creatinine ⩽two-fold of ULN). Exclusion criteria included the central nervous system metastasis; ⩾grade 3 neuropathy; history of earlier cancers within the last 5 years, except for basal cell carcinoma of the skin and *in situ* cancers of the cervix; known human immunodeficiency virus infection; known chronic or acute viral hepatitis; pregnancy or breast-feeding; or a coexisting medical problem of sufficient severity to limit study compliance.

### Treatment and dose modifications

Mapatumumab was provided as a sterile, single-use, lyophilised product. On reconstitution with 5.0 ml sterile water for injection, each vial contained 20 mg ml^−1^ mapatumumab. Once reconstituted, mapatumumab was stored at 2–8°C and infused within 8 h. In cycles 1 and 2 (1 cycle=treatment with mapatumumab on day 1, followed by 14 days of follow-up), each patient received 20 mg kg^−1^ body weight of mapatumumab, administered as an intravenous infusion over 2 h on day 1. In cycle 3 and in subsequent cycles, patients were treated with 10 mg kg^−1^ mapatumumab on day 1. For cycle 3 and for subsequent cycles, the total administration time was reduced to 1 h. The dose and schedule of mapatumumab were selected on the basis of a goal to achieve steady-state concentration of mapatumumab more rapidly than with the previously tested schedules (e.g., 10 mg kg^−1^ every 2 or 3 weeks).

Toxicity was graded according to the National Cancer Institute Common Toxicity Criteria (NCI-CTC; Version 3.0). Dose-modifying toxicities (DMT) were defined as any of the following events if considered related to mapatumumab: grade 3 haematological toxicity lasting ⩽2 weeks or any grade 4 haematological toxicity, grade 3 or greater non-haematological adverse event except nausea/vomiting, diarrhoea, or fatigue for which the following criteria were applied: grade 3 or greater persistent nausea/vomiting in patients who had received optimal medical intervention and/or prophylaxis, grade 3 or greater diarrhoea in patients who received prophylaxis and treatment with anti-diarrhoeal agents, grade 4 fatigue. Mapatumumab dosing was continued at a reduced dose if the DMT recovered to baseline or ⩽grade 1 within 2 weeks of the scheduled treatment. Following a DMT at 20 or 10 mg kg^−1^, the mapatumumab dose in all subsequent cycles was reduced to 10 or 3 mg kg^−1^, respectively. Patients were removed from the trial for the following reasons: disease progression continued with unacceptable toxicities despite optimal treatment or dose reduction, intercurrent illness at the investigator's discretion, withdrawal of consent, non-compliance, lost to follow-up, and pregnancy.

### Assessments

Clinical and laboratory assessments (complete blood count (CBC) with differential, liver function test, urine analysis) were performed every week. Disease assessments were obtained at baseline (within 4 weeks of study entry) and every 6 weeks, or at every three cycles (1 cycle=treatment with mapatumumab on day 1, followed by 14 days of follow-up). Tumour response evaluation was based on the RECIST criteria (Version 1.0; Thérasse *et al*, 2000). Patients were followed up for a minimum of 28 days after the final dose of mapatumumab.

### Pharmacokinetic assessment – plasma pharmacokinetic sampling and assay

Blood specimens were collected in EDTA-coated vacutainer tubes before the administration of mapatumumab, and at the completion of dosing in cycles 1, 2, 3, and 6; at the time of disease assessment on or following the day 8 visit at cycles 3 and 6; and at a minimum of 28 days after the final dose of mapatumumab. If a patient received more than six cycles of mapatumumab therapy, a specimen was collected at the time of disease assessment in every third treatment cycle (i.e., cycles 9, 12, etc.). Blood samples were centrifuged at 1.500 **g** for 10 min immediately after collection, and plasma was stored frozen at −70°C until assayed.

### Determination of plasma mapatumumab concentrations

Plasma samples were analysed for mapatumumab by an enzyme-linked immunosorbent assay (ELISA) using TRAIL-R1:FLAG capture and horseradish peroxidase-labelled anti-human lambda antibody detection. The signal was amplified by the addition of tetramethylbenzidine, followed by absorbance measurement at 450 nm. The assay had a lower limit of quantitation of 100 ng ml^−1^ in undiluted serum.

Plasma mapatumumab concentration data are summarised using mean, median, s.e.m., s.d., coefficient of variation (% CV), geometric mean, 95% confidence interval, and number of patients. Statistical analysis was performed and data appendices were created using either WinNonlin Enterprise (Version 4.1; Pharsight Corp., Mountain View, CA, USA), GraphPad Prism (Version 4.02; GraphPad Software, Inc., La Jolla, CA, USA), or SAS software (Version 8.2; Cary, NC, USA).

The pharmacokinetic (PK) results were inspected for possible effects of age, body weight, gender, study site, and baseline ECOG score. The study was not prospectively designed to assess these factors as potential covariates. Their impact on the plasma mapatumumab concentration results was assessed by comparison of 95% confidence interval between subgroups at corresponding times.

### Pharmacodynamic assessments

Paraffin blocks and/or unstained slides from the original resection or biopsy of the primary tumour and/or other previous biopsies were collected. In addition, if possible, samples of tumour tissue safely amenable to biopsy were obtained at baseline and at one additional time point at least 7 days after receiving mapatumumab in any cycle. The primary use of these samples was the evaluation of TRAIL receptor expression.

### Immunohistochemical staining methods and materials

The immunohistochemical staining method used followed the standardised protocol supplied by DAKO (Glostrup, Denmark) for the investigational-use-only pre-prototype test reagent specific for TRAIL-R1 (Humphreys and Halpern, 2008). Briefly, slides with tissue sections were deparaffinised and hydrated, followed by heat-induced epitope retrieval. A rabbit polyclonal antibody specific for the extracellular portion of human TRAIL-R1 was applied, and a polyclonal rabbit serum Ig was used as negative staining control in parallel. Primary antibody binding signals were amplified using the Envision+ visualisation system (DAKO), and were detected by enzymatic conversion of a substrate chromogen (3′3-diaminobenzidine). Cell lines positive or negative for the target antigen were used as staining controls for each experiment.

Staining was captured using a scoring method developed by DAKO in which distribution and intensity are scored resulting in distinct staining indices (Median Membrane Staining Intensity (M-MSI)) and (Median Cytoplasm Staining Intensity (M-CSI)) for the membrane and cytoplasmic compartments, respectively. Each specimen was scored by a single observer.

### Sample size justification and statistical analysis

A sample size of 30 patients was sufficient to evaluate the response rate of mapatumumab in this population of relapsed or refractory colorectal cancer patients. If no responses are observed, the exact binomial 95% upper confidence limit on the response rate is 9.5%, indicating that such an outcome would provide 95% confidence that the underlying response rate is below this nominal level of activity. If responses are observed, then single-agent activity is indicated and the underlying response rate can be estimated with the precision of approximately ±13%.

### Statistical analysis

Because this study contained no control group and no randomisation to treatment groups, an intention-to-treat analysis was not performed. Instead, the set of patients receiving at least one dose of mapatumumab was included in the as-treated analysis. The primary end point was tumour response as defined by RECIST (Version 1.0). For the purposes of establishing SD, at least 4 weeks from the baseline, measurement had to elapse without PD in order to assign a best response of SD. Progression-free survival, a secondary end point, was defined as time from first treatment to disease progression or death.

## Results

### Patient population

A total of 38 patients were enrolled in this study. Patient characteristics are listed in Table 1. Of the patients, 55% were male and the median age was 63 years. In all, 55% of patients (21 out of 38) had an ECOG performance status of 0 and 45% (17 out of 38) had an ECOG performance status of 1. The patients included in this study were heavily pre-treated. The median number of earlier systemic regimens was 3; 53% (20 out of 38) of patients had received at least three treatment regimens for colorectal cancer previously. Among the five patients who had received more than three regimens earlier, the additional regimens were generally variations of earlier ones. It is noteworthy that nine patients (24%) had previously received cetuximab. Eight patients (21%) had also received radiotherapy earlier. The patients had extensive disease. As reported in the list of target and non-target lesions at baseline, 84% (32 out of 38) of patients had liver lesions, 66% (25 out of 38) had lung lesions, 26% (10 out of 38) had lymph node involvement, 8% (3 out of 38) had spleen involvement, and 26% (10 out of 38) had other lesions.

### Efficacy

A total of 147 cycles of mapatumumab were administered. The median number of cycles administered was 3 (range 1–9). There was one single dose reduction (from 20 to 10 mg kg^−1^ at cycle 2) because of non-haematological toxicity and four dose delays due to non-haematological toxicity and administrative reasons. None of the patients developed an objective tumour response as defined by RECIST (Version 1.0; Table 2). A best response of SD maintained for at least 4 weeks after the baseline assessment was observed for 12 patients (32%), with a median duration of 2.6 months. Modest, short-lived tumour shrinkage of <10% was observed in one patient. In the analysis of PFS, 24 disease progression events were observed (63%), including two deaths due to malignant disease. For all 38 patients, the median PFS was 1.2 months, with a range of 0.0–4.0 months. In 6 of the 38 patients (16%), a PFS of ⩾8 weeks was observed.

### Safety

A summary of treatment-emergent adverse events, regardless of the relationship to mapatumumab, which occurred in >10% of the 38 patients, is shown in Table 3. The most common adverse events were fatigue (37%), nausea (29%), anorexia (24%), and abdominal pain (21%). Most of these events were mild to moderate in severity. The adverse events considered at least possibly related to mapatumumab, which were observed in >5% of patients, comprised diarrhoea (8%), vomiting (8%), and pyrexia (8%). The majority of the related adverse events were mild to moderate in severity.

A total of 11 patients experienced a serious adverse event resulting in hospitalisation. The serious adverse events were considered not to be mapatumumab related by the investigators, with the exception of one event of vomiting. Overall, the most common serious adverse events were ascites, ileus, nausea, fatigue, anorexia, and renal failure, each occurring in two patients.

Summaries of the highest NCI-CTC toxicity grades for selected haematology parameters are presented in Table 4. Leukocytes, neutrophils, and platelets remained within normal limits in >84% of patients while receiving mapatumumab. Among the 38 patients, grade 1 and grade 2 neutropaenia were reported in 4 (11%) and 1 (3%) patients, respectively. Grade 2 (12 out of 38, 32%), grade 3 (3 out of 38, 8%), and grade 4 (1 out of 38, 3%) lymphocytopaenia were observed in 16 out of 38 patients. A total of 6 patients (16%) developed shifts of at least 2 grades from baseline for lymphocytes. There were no significant clinical sequelae reported from these changes in lymphocyte counts. Out of the 38 patients, grade 2 thrombocytopaenia was reported in 2 (5%) patients, grade 1 anaemia was observed in 16 (42%) patients, grade 2 in 10 (26%) patients, and grade 3 in 1 (3%) patient.

No significant haepatotoxicity or renal toxicity was observed. However, at baseline, 22 out of 38 (55%) patients had grade 1 or 2 elevations of AST. Shifts of ⩾2 grades from baseline in AST and/or alanine transaminase were observed in 4 out of 38 (11%) patients. Five patients had grade 3 or 4 alanine transaminase, AST, and/or total bilirubin levels (Table 4). All five of these patients had known liver metastases at study entry and died of progressive disease or its complications.

A total of 152 samples from 38 patients were assayed for immunogenicity to mapatumumab during this study. No anti-mapatumumab antibodies were detected.

### Pharmacokinetics

**Figure 1:**
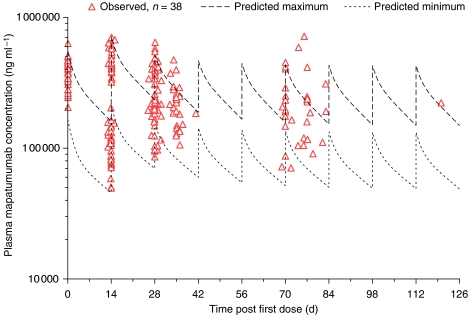
Individual plasma mapatumumab concentrations. Plasma mapatumumab concentrations observed for individual subjects after two 20 mg kg^−1^ mapatumumab intravenous infusion doses given 14 days apart, with 10 mg kg^−1^ mapatumumab intravenous infusion doses given every 14 days thereafter, with the expected minimum to maximum concentration range based on phase 1 study results.

All patients had measurable plasma mapatumumab concentrations after dosing (Figure 1). The plasma mapatumumab concentrations observed in this study were generally within the range of expected concentrations predicted from PK results in previous phase I studies (Tolcher *et al*, 2007; Hotte *et al*, 2008). Gender, age, weight, study site, and baseline ECOG performance status had no apparent effect on mapatumumab exposure. There were no apparent differences in plasma mapatumumab concentrations for patients with stable *vs* progressive disease, or in patients who experienced serious adverse events and those who did not.

### Immunohistochemistry

Paraffin-embedded tissue blocks or 5–10 slides, each with a replicate 3–5 *μ*m tumour tissue section, were provided for 32 of the 38 study patients (84%). A single tissue specimen was provided for 27 patients, and multiple specimens (2–3, collected from different anatomic sites or at different times) were provided for five patients. Most specimens were archival, and were collected 8–64 months before enrolment; one biopsy specimen was collected from a patient on the day of the first mapatumumab administration. All 36 specimens collected were considered to be evaluable after staining. Supplementary Table 1 provides a listing of membrane and cytoplasmic staining results for each patient. The staining index for each compartment covers a possible range of 0–300, with strong staining weighted more heavily than weak staining. As previously observed, staining was variable within and between tumour specimens (Humphreys and Halpern, 2008). Most specimens (28 of 36, 78%) had specific TRAIL-R1 staining on at least 20% of tumour cells. Of these, 20 specimens (20 of 28, 71%) had staining of at least 50% of tumour cells, with staining predominantly cytoplasmic for all but 2 of these 20 specimens (18 of 28, 64%). Three patients did not show detectable staining for TRAIL-R1 in any specimen, and one other patient had a negative specimen and two specimens with weak staining that had been collected at a later date. Most specimens had a mixture of cytoplasmic and membrane staining. However, weak or no membrane staining with stronger cytoplasmic staining was identified for 11 specimens (11 of 28, 39%) from 10 patients, and there was also one example of stronger membrane staining.

Three patients provided specimens collected at different times during their disease course; of these, one set of specimens was consistently negative, one set showed stronger staining earlier in the disease course than at baseline, and one set was initially negative, but demonstrated stronger staining in the specimen collected more recently. In general, the intensity, distribution, and heterogeneity of staining of these specimens were similar to that observed in an independent panel of colorectal carcinoma specimens evaluated using this method (Humphreys and Halpern, 2008).

## Discussion

The primary objective of this phase II clinical study was to assess the tumour response to mapatumumab administered in two loading doses of 20 mg kg^−1^ every 14 days (cycles 1 and 2), followed by maintenance therapy with 10 mg kg^−1^ every 14 days, in patients with refractory advanced colorectal cancer. None of the 38 treated patients developed a response according to RECIST (Version 1.0). Twelve patients had SD of a minimum duration of at least 4 weeks. Modest, short-lived tumour shrinkage of <10% was observed in one patient. The median PFS for the 38 study patients was 1.2 months. Hence, when administered as a single agent, mapatumumab did not demonstrate the ability to shrink tumours or generate a clinically meaningful response in this population of heavily pre-treated refractory colorectal cancer patients. Similar results were also reported with mapatumumab in patients with pre-treated advanced non-small-cell lung cancer (Greco *et al*, 2008). However, two complete and one partial responses were reported in patients with relapsed or refractory follicular lymphoma receiving mapatumumab (Younes *et al*, 2005), demonstrating that single-agent mapatumumab has activity at these doses.

Resistance to TRAIL receptor (TRAIL-R)-mediated signalling in colorectal cancer has been observed in both colorectal cell lines and *ex vivo* transplants of patient tumours grown in mouse xenografts. Resistance can be mediated at the cell surface through changes in receptor expression levels and intracellular proteins that impinge on both the extrinsic and intrinsic apoptotic pathway.

Several pieces of evidence support the observation that resistance to TRAIL-R signalling increases as tumours progress from benign to advanced, metastatic disease. High levels of TRAIL decoy receptor expression are associated with poor prognosis and resistance to 5-fluorouracil (Granci *et al*, 2008) or oxaliplatin (Toscano *et al*, 2008) and to increased levels of anti-apoptotic proteins survivin and XIAP in metastatic colorectal cell lines (Huerta *et al*, 2007). The importance of TRAIL-R levels in determining sensitivity or resistance is validated by changes in the TRAIL-R expression in response to chemotherapy, radiation, or steroids that are associated with increased responsiveness (Gong *et al*, 2006; Kouhara *et al*, 2007; Oikonomou *et al*, 2007).

Overexpression of the anti-apoptotic protein, FLIP-L, has been demonstrated to be a prognostic factor in colorectal cancer (Heijink *et al*, 2007; Korkolopoulou *et al*, 2007; Ullenhag *et al*, 2007). Experimental manipulation of FLIP (Wilson *et al*, 2007) and XIAP (Toscano *et al*, 2008; Connolly *et al*, 2009; Wilson *et al*, 2009) has confirmed their critical role in mediating resistance against TRAIL-R-dependent apoptosis. High levels of the anti-apoptotic proteins Bcl-xL (Schulze-Bergkamen *et al*, 2008), BAG-1 (Clemo *et al*, 2008), AKT, and Bax (Niyazi *et al*, 2009) also mediate TRAIL resistance in colorectal cell lines.

In pre-clinical studies, mapatumumab activity is more impressive when combined with chemotherapeutic agents (Pukac *et al*, 2005). In colorectal cancer cell lines COLO205, HCT116, and SW480, the combination of mapatumumab and 5-fluorouracil, topotecan, or irinotecan demonstrated significantly higher cytotoxicity than either chemotherapy or mapatumumab alone, suggesting a potential for synergy. In an SW480 colorectal cancer xenograft model, administration of mapatumumab, in combination with topotecan, resulted in significant inhibition, compared with single-agent topotecan. Similarly, enhanced cell killing was observed in NSCLC (H460), breast (MDA-MB-231), lymphoma (ST486), and ovarian (SKOV3) cancer models when mapatumumab was combined with different chemotherapeutic agents (Pukac *et al*, 2005). Potential mechanisms that could explain the pre-clinical synergy between mapatumumab and chemotherapy include increased expression of death receptors, increased expression of pro-apoptotic Bax/Bak family members, and decreased expression of anti-apoptotic Bcl-2/Bcl-XL family members and IAPs (Thorburn *et al*, 2008). Redistribution and clustering of death receptors in cells treated with chemotherapeutic agents and modulation of death-inducing receptor complex components have also been reported (Elrod and Sun, 2008; Yu and Shi, 2008).

Hence, after this phase II monotherapy study, a logical step would be to explore mapatumumab in combination with chemotherapy agents in patients with advanced colorectal cancer. In this regard, other biological agents, notably bevacizumab, had limited activity in colorectal cancer as a single agent but improved survival when combined with standard chemotherapy regimens (Hurwitz *et al*, 2004).

A secondary objective of this study was to evaluate the safety and tolerability of mapatumumab in this heavily pre-treated patient population. In general, mapatumumab was well tolerated. The toxicity profile of mapatumumab observed in this study was consistent with that in single-agent phase I studies (Tolcher *et al*, 2007; Hotte *et al*, 2008). Most adverse events reported to be mapatumumab related were mild to moderate in severity. Mapatumumab did not seem to markedly affect haematological parameters other than lymphocyte counts. In this study, 42% of patients experienced lymphopaenia, consistent with other previously published single-agent mapatumumab studies (Tolcher *et al*, 2007; Greco *et al*, 2008; Hotte *et al*, 2008). None of these transient lymphocytopaenias were associated with clinically relevant infections, but subsequent studies of mapatumumab should evaluate this further, for example, by characterising lymphocyte subsets in patients who experience lymphocytopaenia, especially as TRAIL targets certain lymphocyte subsets (Janssen *et al*, 2005).

Importantly, in this study, mapatumumab exhibited no relevant haepatotoxicity, an *a priori* concern in the light of pre-clinical studies of TRAIL receptor agonists (Yin and Ding, 2003; Mori *et al*, 2004; Zheng *et al*, 2004). The safety profile of single-agent mapatumumab administered at doses of up to 20 mg kg^−1^ every 14 days in heavily pre-treated advanced colorectal cancer patients was quite favourable.

The heterogeneity of immunohistochemistry staining for TRAIL-R1 observed in this study was consistent with that of earlier observations. The lack of responses to single-agent mapatumumab precluded any evaluation of association between receptor expression and clinical outcome.

Early safety results from ongoing clinical trials of mapatumumab with several chemotherapy regimens are promising (Leong *et al*, 2009; Mom *et al*, 2009). Results reported to date have shown that the combinations tested (i.e., paclitaxel, carboplatin plus mapatumumab, as well as gemcitabine, cisplatin plus mapatumumab) were safe and well tolerated. On the basis of the results of the current study, as well as on the pre-clinical evidence of synergy in combination with commonly used agents in colorectal cancer, and the encouraging safety and efficacy data reported in the ongoing clinical trials of mapatumumab combined with full-dose chemotherapy, clinical trials of mapatumumab in combination with standard chemotherapy agents in patients with advanced colorectal cancer are warranted.

## Figures and Tables

**Table 1:** Patient characteristics

	**Number of patients (%) (*n*=38)**
*Gender*	
Male	21 (55)
Female	17 (45)
	
*Age (years)*	
Median	63
Range	39–84
	
*ECOG performance status*	
0	21 (55)
1	17 (45)
	
*Number of previous systemic regimens*	
Median	3
Range	1–6
1 previous regimen	4 (11)
2 previous regimens	14 (37)
3 previous regimens	15 (40)
4 previous regimens	3 (8)
5 previous regimens	1 (3)
6 previous regimens	1 (3)

**Table 2:** Summary of best response

	**Number of patients (%) (*n*=38)**
*Evaluable*	35 (92)
CR	—
PR	—
SD	12 (32)
95% CI	18–49
Median duration of SD (months)	2.6
95% CI	2.6–2.8
PD	23 (61)
95% CI	43–76
	
*Not evaluable*	3 (8)
Early death^a^	1 (3)
Insufficient data	2 (5)

Abbreviations: CI=confidence interval; CR=complete response; PD=progressive disease; PR=partial response; SD=stable disease.

a Death due to progressive disease.

**Table 3:** Summary of the most frequent treatment-emergent^a^ adverse events (*n*=38)

	**All grades, *n* (%)**	**Grade 1, *n* (%)**	**Grade 2, *n* (%)**	**Grade 3, *n* (%)**	**Grade 4**
Fatigue	14 (37)	8 (21)	9 (11)	2 (5)	—
Nausea	11 (29)	3 (8)	4 (11)	4 (11)	—
Anorexia	9 (24)	5 (13)	3 (8)	1 (3)	—
Abdominal pain	8 (21)	3 (8)	1 (3)	4 (11)	—
Diarrhoea	6 (16)	3 (8)	3 (8)	—	—
Dyspnea	6 (16)	1 (3)	1 (3)	4 (11)	—
Peripheral oedema	6 (16)	3 (8)	2 (5)	1 (3)	—
Vomiting	6 (16)	2 (5)	1 (3)	3 (8)	—
Insomnia	5 (13)	3 (8)	2 (5)	—	—
Pyrexia	5 (13)	3 (8)	2 (5)	—	—
Ascites	4 (11)	1 (3)	1 (3)	2 (5)	—
Back pain	4 (11)	2 (5)	2 (5)	—	—
Constipation	4 (11)	2 (5)	1 (3)	1 (3)	—
Cough	4 (11)	1 (3)	3 (8)	—	—

a Regardless of study drug relationship.

**Table 4:** Selected haematologic, hepatic and renal toxicities (according to NCI-CTC criteria) in all mapatumumab-treated patients (*n*=38)

	**Grade 0, *n* (%)**	**Grade 1, *n* (%)**	**Grade 2, *n* (%)**	**Grade 3, *n* (%)**	**Grade 4, *n* (%)**
Leukocytes	32 (84)	5 (13)	1 (3)	—	—
Neutrophils (ANC)	33 (87)	4 (11)	1 (3)	—	—
Lymphocytes	22 (58)	—	12 (32)	3 (8)	1 (3)
Platelets	36 (95)	—	2 (5)	—	—
Haemoglobin	11 (29)	16 (42)	10 (26)	1 (3)	—
AST	12 (32)	13 (34)	8 (21)	4 (11)	1 (3)
ALT	21 (55)	11 (29)	5 (13)	—	—
Total bilirubin	29 (77)	3 (8)	5 (13)	—	1 (3)
Creatinine	24 (63)	8 (21)	5 (13)	—	1 (3)

Abbreviations: ANC=absolute neutrophil count; ALT=alanine transaminase; AST=aspartate transaminase.
