# Supplementary material for: Phase II trial of mapatumumab, a fully human agonistic monoclonal antibody that targets and activates the tumour necrosis factor apoptosis-inducing ligand receptor-1 (TRAIL-R1), in patients with refractory colorectal cancer
Source: Br J Cancer. 2010 Jan 12;102(3):506–12. doi: 10.1038/sj.bjc.6605507 (PMC2822942; doi:10.1038/sj.bjc.6605507)
Supplement: Supplementary Table 1 [file 6605507x1.doc]

**SUPPLEMENTAL TABLE 1 – Membrane (MSI) and Cytoplasmic (CSI) Staining Results for each Patient with IHC Specimen (n=32)**

| **Subject no.** | **Approximate collection**  **interval*** | **Mean MSI** | **Mean CSI** | **Best response** |
| --- | --- | --- | --- | --- |
| 1 | 11 months | 20 | 170 | PD |
| - | 11 months | 20 | 60 | - |
| 2 | 15 months | 0 | 100 | PD |
| 3 | 8 months | 0 | 40 | PD |
| 4 | 15 months | 0 | 40 | PD |
| 5 | 64 months | 0 | 0 | PD |
| 6 | 18 months | 30 | 100 | Not Evaluable |
| 7 | 15 months | 30 | 80 | SD |
| 8 | 36 months | 140 | 40 | SD |
| 9 | 50 months | 30 | 90 | PD |
| 10 | 10 months | 10 | 60 | PD |
| 11 | 20 months | 30 | 120 | SD |
| 12 | 45 months | 60 | 60 | SD |
| 13 | 12 months | 80 | 150 | SD |
| 14 | 21 months | 10 | 20 | SD |
| 15 | 16 months | 0 | 100 | PD |
| 16 | 18 months | 60 | 100 | PD |
| 17 | 32 months | 10 | 70 | PD |
| 18 | 28 months | 0 | 10 | SD |
| 19 | 37 months | 0 | 10 | SD |
| 20 | 9 months | 20 | 80 | Not Evaluable |
| 21 | 19 months | 20 | 30 | PD |
| 22 | 15 months | 30 | 70 | PD |
| 23 | 20 months | 30 | 70 | PD |
| 24 | 21 months | 30 | 70 | PD |
| 25 | (Baseline) | 10 | 10 | PD |
| - | 36 months | 40 | 70 | - |
| 26 | 25-37 months | 0 | 0 | SD |
| - | 42 months | 0 | 0 | - |
| 27 | 31 months | 0 | 0 | PD |
| 28 | 19 months | 10 | 50 | SD |
| - | 39 months | 0 | 0 | - |
| 29 | 14 months | 40 | 60 | PD |
| 30 | 22 months | 30 | 130 | PD |
| 31 | 18 months | 70 | 190 | PD |
| 32 | 14 months | 90 | 10 | SD |

***Defined by time between collection of specimen and enrolment in the clinical trial.**
